# Neonatal Colonisation Expands a Specific Intestinal Antigen-Presenting Cell Subset Prior to CD4 T-Cell Expansion, without Altering T-Cell Repertoire

**DOI:** 10.1371/journal.pone.0033707

**Published:** 2012-03-19

**Authors:** Charlotte F. Inman, Georgina M. Laycock, Louisa Mitchard, Ross Harley, James Warwick, Rachel Burt, Pauline M. van Diemen, Mark Stevens, Mick Bailey

**Affiliations:** 1 School of Clinical Veterinary Science, University of Bristol, Langford, Bristol, United Kingdom; 2 Institute for Animal Health, Compton, Newbury, Berkshire, United Kingdom; McGill University, Canada

## Abstract

Interactions between the early-life colonising intestinal microbiota and the developing immune system are critical in determining the nature of immune responses in later life. Studies in neonatal animals in which this interaction can be examined are central to understanding the mechanisms by which the microbiota impacts on immune development and to developing therapies based on manipulation of the microbiome. The inbred piglet model represents a system that is comparable to human neonates and allows for control of the impact of maternal factors. Here we show that colonisation with a defined microbiota produces expansion of mucosal plasma cells and of T-lymphocytes without altering the repertoire of alpha beta T-cells in the intestine. Importantly, this is preceded by microbially-induced expansion of a signal regulatory protein α-positive (SIRPα^+^) antigen-presenting cell subset, whilst SIRPα^−^CD11R1^+^ antigen-presenting cells (APCs) are unaffected by colonisation. The central role of intestinal APCs in the induction and maintenance of mucosal immunity implicates SIRPα^+^ antigen-presenting cells as orchestrators of early-life mucosal immune development.

## Introduction

The importance of microbial colonisation of the gut in immune development is well established. Early studies in germ-free (GF) mice demonstrated that colonisation was critical for the induction of IgA- producing plasma cells in the intestine [1] and drove CD4+ cell expansion [2]. Studies in rabbits have demonstrated a similar picture: follicular development was arrested in rabbit appendices that were ligated to prevent colonisation [3] and the appendices of GF rabbits contain reduced numbers of lymphocytes [4]. More recent studies have shown that this development is qualitative as well as quantitative: colonisation produces a wider B-cell repertoire in pigs and results in differentiation of CD4 T helper (Th) cell subsets in mice [5]–[7]. However, it appears that simple ‘colonisation’ is not enough to generate a fully effective immune system capable of recognising and eliminating pathogen whilst not responding to self, food and commensal bacterial antigen. In fact, epidemiological studies in humans (linking microbiota and the incidence of allergy) have indicated that the nature of colonisation in the post-natal period is important in determining the character of the immune system that subsequently emerges [8], [9]. In order to fully investigate these observations, it is necessary to use a reductionist rather than holistic approach to experimental design, colonising GF animals with a specifically defined microbiota and measuring the effect on immune development. These studies have been elegantly performed in rodent models and have identified specific species of bacteria that appear to be responsible for the development of specific arms of the immune response. For example, *Bacteroides fragilis* has been shown to be important in the development of FoxP3^+^ T cells in the intestine and segmented filamentous bacteria (SFB) have been shown to drive the appearance of Th17 cells in the intestinal lamina propria [10], [11]. This data confirms that the type of colonisation may be as important as the colonisation event itself.

The antigen-presenting cell (APC) is central to the induction and maintenance of immune responses. This is particularly clear in the intestine where APCs can be observed directly interacting with the microbiota [12], [13] and with T cells, not only in the lymph nodes but also in the mucosa [14]. Work in mice has identified functionally distinct populations of dendritic cells (DCs) in the intestinal mucosa, distinguished by their expression of CD103, CX_3_CR1 and CD11b [15], [16] and it appears that these different intestinal APC subsets are important in generating different types of immune response [17], [18]. There are also distinct populations of intestinal APC in the pig (based on expression of CD16, CD11R1 and SIRPα [14], [19], [20]) and it appears that these may also be functionally different [20]. Whilst this suggests that the presence of different APC subsets may be important across species, it remains to be seen whether it is possible to identify direct functional equivalents for all of the recognised mouse intestinal APC subsets in other animals. For example, although the same monoclonal antibody recognizes human CD11b and pig CD11R1 [21], there are significant differences in the distribution of the target molecules [22], indicating that they are not necessarily functionally equivalent in mouse, human, and pig. Nevertheless, it appears that expression of SIRPα may, in fact, identify APCs with a similar phenotype between species. For example, SIRPα expression has been shown to be correlated with the ability of APCs to migrate to the lymph nodes in pigs and mice [20], [23]. In humans, sheep, cattle and rats, SIRPα expression distinguishes functionally different APC subsets [24]–[28]. Thus, relative expression of SIRPα by intestinal APCs is likely to be important in determining immune function in the gut. In relation to the effects of the microbiota on intestinal APCs, SIRPα has not been examined. However, work in adult mice has demonstrated that colonisation preferentially expands the CX_3_CR1^+^ APC subset in the colon [17]. We have previously demonstrated a selective effect of different microbiota (associated with high hygiene and low hygiene rearing environments) on myeloid APCs in the neonatal pig intestine but not on endothelial APCs [29]. However, generally, data on the effects of colonisation on APC subsets in the small intestine, and on neonates, is sparse. This is despite the well documented importance of early life colonisation and subsequent immune development on the ability to mount effective responses later in life.

Whilst mucosal APCs are a core part of immunity in the intestine, T cells are one of the ‘workhorses’ of the adaptive immune response that, as a population, are capable of responding to a massive range of antigens due to the variability and promiscuity of the T cell receptors expressed by different clones. The hypervariable complementarity determining region 3 (CDR3) of the T cell receptor (TCR) interacts with the major histocompatibility complex (MHC) plus peptide on the surface of APCs. Activation of T cells with a specific antigen may lead to expansion of a few specific T cell clones. By using spectratyping to analyse CDR3 lengths of T cells within the intestinal mucosal population, it is possible to perceive whether one or more dominant CDR3 lengths are present and, thus, whether there is likely to have been antigen-specific recruitment of T-cells to the mucosa. For example, spectratype studies in rats have shown that T-cells in the intestine have a restricted, oligoclonal repertoire [30]. By comparing the distributions of CDR3 lengths in animals colonised with a defined flora to the distributions in GF animals, it is possible to determine whether colonisation alters T cell repertoire in the gut. This is an important matter for investigation, as changes in repertoire associated with colonisation may have a major impact on gut immune function. For example, although induction of a broad repertoire of T-cell clones following colonisation of the gut may subsequently promote host defence against an extremely wide range of pathogens, competition for resources between ‘useful’ T cell clones and T cells with irrelevant TCRs may mean that an alternative strategy, in which only a limited cohort of ‘useful’ T cell clones is induced, proves to be more effective in defending against a (restricted) range of pathogens. Notably, in relation to the effects of intestinal colonisation on B-cells, previous studies in GF pigs and mice have, in the majority, indicated that B cell expansion and IgA production after colonisation is primarily antigen non-specific [31]–[34]. Whilst spectratyping does not allow for identification of expansion of specific T cell clones in response to specific antigens, it has the advantage of generating a general overview of the effects of colonisation on the diversity of the TCR repertoire.

In order to control for maternal effects, it is necessary to examine immune development in neonates reared away from the mother, and without transferred antigen or antibody. As a consequence of this, these types of studies are technically difficult in rodents, where significant transfer occurs *in utero* [35]. As a solution to this problem, we have used a piglet GF model where highly inbred Babraham piglets are derived by Caesarean section and housed individually under sterile conditions. The use of the pig model reduces variation associated with maternal factors and allows for controlled colonisation with a defined microbiota. The pig model is particularly appropriate for the study of the impact of intestinal colonisation since no placental transfer of macromolecules takes place [35], [36], and since intra-individual stability and inter-individual variability of the microbiota are more comparable between humans and pigs than between humans and mice [37]. Pigs have comparable digestive physiology to humans and full genome studies have demonstrated fewer differences between humans and pigs that between humans and mice [38]. In addition, as in human infants, the neonatal piglet has a poorly developed mucosal immune system [39]. Whilst the GF pig model has been well utilised over a number of years for these reasons, a further advantage of the model described in this study is in the use of inbred, Babraham pigs, which reduces the variation associated with genetic factors.

We set out to investigate the effects of colonisation with a defined microbiota on development of the adaptive mucosal immune system in a neonatal pig model. To this end, we have developed a defined microbiota and used this to colonise neonatal piglets in the first 24 hours of life, to reflect the situation in human infants. We used quantitative immunohistology to examine the effects of colonisation on B and T lymphocyte cell numbers. On finding, in concordance with previous studies, that colonisation increased the numbers of these cells in the mucosa, we used spectratype analysis of 21 *TRβ*V groups/subgroups (see Table S1) to examine whether it also altered T cell repertoire diversity. Interestingly, colonisation had no apparent effect on TCR Vβ repertoire, diversity suggesting that the expansion, whilst driven by colonisation, did not solely involve expansion of T-cells specific for the microbiota. Given our previous studies on intestinal APC subsets and, more importantly, the observation that in adult mice, a specific subset is affected by colonisation, we were keen to examine the effects of colonisation on APC subsets in the neonate. Strikingly, quantitative immunohistology showed that colonisation in neonates specifically increased the SIRPα+ APC subset.

## Materials and Methods

### Ethics statement

All animal experiments were conducted in accordance with the Animal (Scientific Procedures) Act 1986 (licence 30/2485) with the approval of the Institute for Animal Health Ethical Review Committee.

### Animals and housing

Four separate experiments (see Table 1) were performed in which inbred Babraham piglets were derived by Caesarean section into a sterile chamber. Within two hours, the animals were transferred to individual sterile housing (Bell Isolation Systems, UK), or killed for day 0 time point sampling as described below. Piglets were fed on tinned, autoclaved evaporated milk (Tesco PLC, UK) and had free access to sterile water.

**Table 1:** Numbers and ages of GF and colonised pigs in each experiment.

Experiment	Age of pigs (days)	No. GF pigs	No. colonised pigs
1	0	5	N/A
2	5	3	3
3	21	3	4
4	21	1	2

Four experiments were performed with between five and seven pigs in each. Pigs were euthanised and samples taken either at 0 (1 experiment), 5 (1 experiment) or 21 (2 experiments) days of age.

### Microbial flora

The colonisation microbiota consisted of a cocktail of bacteria comprising *Lactobacillus amylovorus* DSM16698, *Clostridium glycolicum* and *Parabacteroides* sp. (Altered Schaedler Flora (ASF) 519). The ideal attributes we considered when deciding on the composition of the defined microbiota were: how practical species were to grow and administer without contamination; whether they could be administered immediately after derivation without causing disease; whether they produced consistent, prolonged colonization of at least one intestinal region; whether they produced measurable effects on the host immune system. Host immune development is likely to reflect exposure to a range of microbial associated molecular patterns (MAMPs), expressed by different bacterial species: hence, the greater the diversity of bacteria selected, the more likely it would be that the microbiota would contain a wider range of MAMPs. Therefore, the selection of species for this defined microbiota was based on the use of one bacterial strain from each of the four most frequently identified phylogenetic groups identified in the ileum, caecum and colon in 12–18 week old pigs [40]. The groups and the chosen species were as follows:

1. *Eubacterium* and relatives: *Roseburia intestinalis*, a porcine isolate [41] able to grow in media containing a wide range of metabolic carbohydrate substrates, including many simple saccharides [42]
2. *Clostridium* and relatives: *Clostridium glycolicum* identified in the luminal content of unweaned pigs (unpublished data)
3. *Bacillus*-*Lactobacillus*-*Streptococcus* (BLS) subdivision: *Lactobacillus amylovorus* DSM 16698^T^ (previously known as *L. sobrius* [43], a porcine isolate, found in unweaned pigs [44] which has been shown to be protective against *in vivo* challenge with enterotoxigenic *E. coli*, in conventionally colonized pigs [45].
4. *Cytophaga*-*Flexibacter*-*Bacteroides* (CFB) group: *Parabacteroides* sp. (ASF519) [46] which was shown to give reliable colonization of the large intestine in previous experiments (data not shown).

A preliminary experiment demonstrated that *C. glycolicum*, *L. amylovorus* and ASF 519 colonised the length of the intestinal tract of 3 GF pigs (Proximal jejunum, distal jejunum, terminal ileum, caecum and colon), whereas *R. intestinalis* failed to colonise any of these sites in any piglet (data not shown). Therefore, *R. intestinalis* was not added to the bacterial cocktail in the experiments described here.

All bacteria were cultured on pre-reduced agar plates, in a MK3 Anaerobic Work Station (Don Whitley Scientific Limited, UK) at 36.5°C or incubated at 37°C in a 2.5 L anaerobic jar containing an AnaeroGen sachet (Oxoid Limited, UK). Once strains were considered pure, bacterial colonies were anaerobically transferred into pre-reduced, sterile, anaerobic 50 ml vials containing 15 mL of broth with 0.5 g/L sodium sulphide as a reducing agent and 0.01% wt/vol resazurin as an oxygen indicator. *L. amylovorus* was cultured using de Man Rogosa Sharpe (MRS) agar and broth (Oxoid Limited). All other bacterial species were cultured on pre-reduced Schaedler agar and broth (BD Biosciences, UK). After transfer, vials were cultured for a further 24–48 hours, dependent on bacterial species, until broth was turbid. Broth cultures were checked for contaminants by light microscopy and by aerobic and anaerobic culture on Schaedler agar and blood agar plates. Prior to administering the microbiota to piglets, the identity of each bacterial strain in broth culture was confirmed by DNA sequence analysis of 16S rDNA.

### Experimental setup

Half of each group of piglets was colonised orally at 0, 1 and 2 days of age with the cocktail of bacteria (Table 1). The other half remained germ-free. Swabs taken from surgical sites during the Caesarean section and weekly rectal swabs from piglets were plated onto blood agar for aerobic and anaerobic culture for 48 hours to check for bacterial contaminants.

Any contaminated animals were removed from the study. Colonisation with individual species within the microbiota was confirmed by species-specific 16S rDNA-specific polymerase chain reaction (PCR). This demonstrated that all piglets were colonised with all the bacterial species in proximal and distal jejunum, caecum and colon.

At 0, 5 and 21 days of age (Table 1), animals were killed with an overdose of sodium pentabarbitone and tissues were collected for immunohistology and RNA extraction.

### Immunohistology

Sections of jejunum without Peyer's patches were collected, snap-frozen and prepared for immunohistology as described previously [47]. The following anti-pig monoclonal antibodies (mAb) were used: immunoglobulin A (IgA) (clone K61.1B4), IgM (clone K52.1C3), CD4 (clone MIL17), MHCII DR (clone MSA-3), CD16 (clone G7), CD11R1 (clone MIL4), SIRPα (CD172)-FITC (clone 74-22-15; Serotec, UK) capillary endothelium (clone MIL11). Binding was detected with goat anti-mouse IgG1 FITC or TRITC; goat anti-mouse IgG2b FITC or TRITC (Southern Biotechnology, UK); goat anti-mouse IgG2a AlexaFluor 633 (Invitrogen, UK), biotinylated goat anti-mouse IgG1 (Southern Biotechnology) and biotinylated rat anti-nouse IgE (Southern Biotechnology). Where directly conjugated SIRPα was used, it was added with mouse serum after staining with the other primary and secondary antibodies. Biotinylated anti-sera were detected with 7-amino-4-methylcoumarin-3-acetic acid-Avidin-D (Vector Laboratories, UK).

### Image analysis

Ten images/tissue of each stain were captured using a Leica DMR-A fluorescence microscope fitted with appropriate single colour filters, a Hamamatsu Orca-ER camera (Hamamatsu, UK) and Q-fluoro software (Leica, Germany). Images were analysed as described previously using *ImageJ* software [47]. Briefly, levels of background staining (threshold levels) in all colour channels were obtained from negative control slides (prepared in conjunction with each sample) using the *ImageJ* macro ‘*multiplecolourbackgrounds*’. These were then applied to each positively stained image so that all pixels above threshold were set to their appropriate colour and all those below were set to black. This technique has been previously optimised to minimise variation between samples [47]. The number of pixels positive for each colour were then analysed using the *ImageJ* macro ‘*multiplecolouranalysis*’. This data was then expressed as the proportion of pixels positive for each colour in the whole image.

### RNA extraction, analysis and reverse transcription

Sections of spleen, proximal jejunum and distal jejunum tissue (without Peyer's patches) were collected into RNALater (Ambion Ltd., UK) according to manufacturer's instructions and stored at -70°C. Tissues were homogenized with a stainless steel bead using a TissueLyser (Qiagen, UK). RNA was extracted from tissue samples using the SV RNA Total Isolation System (Promega, UK) according to manufacturer's instructions, and concentration and quality was assessed using the Experion Automated Electrophoresis system (Bio-Rad, UK). Reverse transcription was performed using random hexamers and the Improm II Reverse Transcription system (Promega). cDNA was diluted to a final working volume of 150 µl and stored at −20°C.

### Porcine T cell receptor V beta (pTRβV) Spectratype PCR

Phylogenetic analysis of 363 *TRβV* nucleotide sequences (unpublished data) allowed the identification of 20 *TRβV* groups. Nineteen of these contained clones corresponding to the 19 pig *TRβV* groups named by Butler et al [48], using the IMGT designation. The final group contained a sequence (GenBank **AB079527**) with high homology to human *TRβV*24 transcripts: this sequence was recently confirmed as porcine *TRβV*24 [49]. Within *TRβV*12S, one sequence (GenBank **AY690854**) had a significantly lower identity with other *TRβV* sequences in the group. This sequence was, therefore, designated *TRβV*12-AS: a subgroup of *TRβV*12S.

For each cDNA sample, *TRβV* group/subgroup-specific PCR products were generated using sense primers specific for one of 20 *TRβV* groups, or 1 *TRβV* subgroup, and a universal *Cβ*-region antisense primer (Table S1) labelled with 5′ FAM, HEX or TET. PCR was performed using a PTC-200 DNA Engine (MJ Research, Massachusetts). Each PCR mixture comprised 2 µl template in 25 µl 1× HotStartaq mastermix (Qiagen, UK) containing 200 nM each of sense *TRβV*-specific primer and labelled antisense *Cβ*-region primer and 3.0 mM MgCl_2_. PCR conditions were 95°C for 15 minutes, then 40 cycles of 15 s at 95°C, 15 s at 60°C, and 30 s at 72°C and 5 minutes at 72°C. PCR products from positive and negative control samples were visualised on a 2% agarose gel stained with ethidium bromide prior to sample purification. PCR product purification was performed using the Nucleospin Multi-96 Extract PCR clean-up kit (Macherey-Nagel, Germany). Fluorimetry of the purified PCR products was performed using the quantitative plate read function on the MX3005P QPCR system (Stratagene, UK). A standard curve was constructed for the fluorochromes FAM, HEX and TET from a two-fold serial dilution from 10 nM to 0.15625 nM using the universal *Cβ*-region antisense primer labelled with each fluorochrome diluted in AE buffer, and a buffer only negative control. Approximate concentrations of fluorescently-labelled PCR products were derived and used to determine the amount of purified PCR product for subsequent use.

### Spectratype analysis

For generation of spectratype electropherograms, purified PCR products were appropriately mixed (different sizes and fluorochromes) and processed using a MegaBACE 1000 DNA analysis system (Amersham, UK) at the University of Bristol Transcriptomics Facility. Electropherograms generated for each well were analysed using the MegaBACE Genetic Profiler v 1.5 (Amersham). Each electropherogram peak analysed was verified as within the anticipated bp range. Peaks which were not differentiable from background fluorescence (<70 relative fluorescence units (rfu)) or which were >1 bp away from the expected size were rejected. Peak rfu (heights) were standardised by conversion to a proportion of the total peak rfu for a given spectratype electropherogram [30].

### Statistics

Data were analysed using SPSS 18.0 software (SPSS Inc., Chicago). Immunohistology images were analysed using analysis of variance ANOVA with post-hoc testing using least significant difference and with proportion positive area as the outcome variable and treatment (colonisation status) and age as explanatory variables. Pig was included in the model as a covariate. Where appropriate, a Bonferroni correction was applied for multiple tests.

For spectratyping, analysis was performed using principle component analysis (PCA) followed by a univariate general linear model (GLM) with post-hoc testing using least significant difference and hierarchical cluster analysis. PCA was performed on all *TRβV* groups/subgroup at once for a maximum of 25 iterations for convergence using an unrotated factor solution. All components analysed had Eigenvalues greater than 1. Hierarchical cluster analysis was performed using Wards method with squared Euclidean distances [30]. Unless otherwise stated, significance was defined as p≤0.05.

## Results

### Microbial colonisation expands mucosal plasma cells and CD4 T-cells

Whilst other studies in pigs have demonstrated an increase in mucosal plasma cells and lymphocytes on colonisation [31], [32], [39], we wanted to study the effects of colonisation with our microbiota on these cells in more detail. Therefore, we examined the effects of colonisation on lymphoid cells at 5 and 21 days of age.

**Figure 1:**
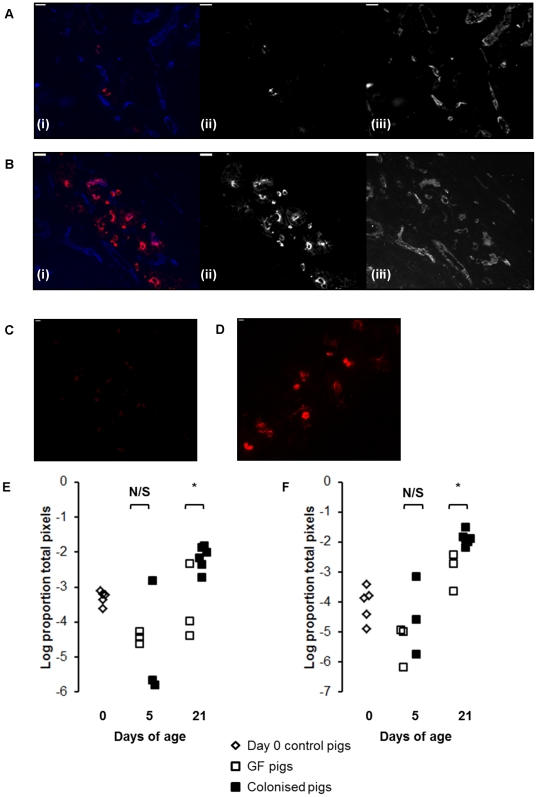
Colonisation expands IgM and IgA-producing mucosal plasma cells. (A) IgA staining in jejunal mucosa from a GF pig and (B) a colonised pig at 21 days of age. Sections were stained with antibodies to IgA (red) and capillary endothelium (MIL11; in blue). In both (A) and (B), (i) shows the two-colour image; (ii) shows the original greyscale image for the red channel (IgA); (iii) shows the original greyscale image for the blue channel (endothelium). Scale bar represents 10 µm. (C) IgM staining in jejunal mucosa from a GF pig and (D) a colonised pig at 21 days of age. Sections were stained with antibodies to IgM (red). Scale bar represents 10 µm. (E) Area of IgM and (F) IgA staining in jejunal sections from piglets at birth (open diamonds) and GF piglets (open squares) and colonised piglets (black squares) at 5 and 21days of age (see Table 1 for numbers of piglets in each experiment). * p<0.01.

Since Ig-producing cells reside in the intestinal crypts in the pig small intestine, we examined the crypt regions of jejunal sections from GF and colonised pigs using immunofluorescent labelling to look for the effects of colonisation on Ig-producing cells. This showed a clear increase in both IgM and IgA-producing plasma cells associated with colonisation (Figure 1A and B). Quantitative analysis of the images showed that this effect was statistically significant by 21 days of age and not at 5 days (Figure 1C and D; p<0.01).

**Figure 2:**
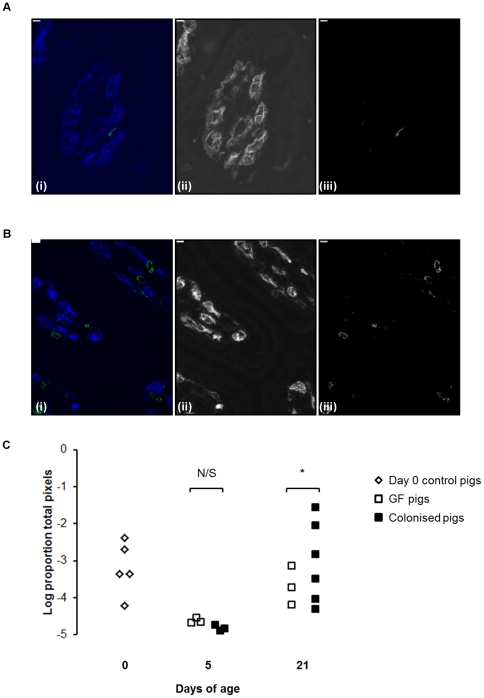
Colonisation expands mucosal CD4^+^ T cells by 21 days of age. (A) CD4 staining in jejunal mucosa from a GF pig and (B) a colonised pig at 21 days of age. Sections were stained with antibodies to CD4 (green) and capillary endothelium (MIL11; in blue). In both (A) and (B), (i) shows the two-colour image; (ii) shows the original greyscale image for the blue channel (endothelium); (iii) shows the original greyscale image for the green channel (CD4). Scale bar represents 10 µm. (C) Area of CD4+ T cell staining in jejunal sections from piglets at birth (open diamonds) and GF piglets (open squares) and colonised piglets (black squares) at 5 and 21 days of age (see Table 1 for numbers of piglets in each experiment). *p<0.05.

A similar picture could be seen in the CD4 T-cell compartment, colonisation producing an increase in CD4 T-cells (figure 2A and B). Similar to plasma cells, this increase was only significant by 21 days (figure 2C; p<0.05). As demonstrated previously, CD4 T cells could be seen co-localising with MHCII+ cells in the lamina propria (data not shown) [14].

### TCR V beta is unaffected by colonisation-induced T-cell expansion

**Figure 3:**
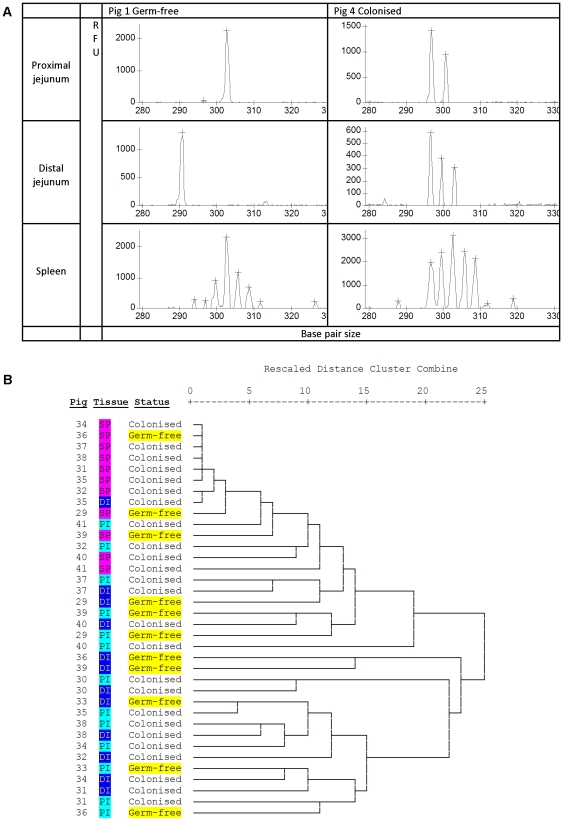
Repertoire is not altered on expansion of mucosal CD4 T-cells. (A) Representative spectratype electropherograms of *TRβV*29 for a germ-free pig, and a colonised pig from the same litter of piglets. In general, samples from the spleen demonstrate a more Gaussian electropherogram distribution than intestinal samples. RFU: relative fluorescence units, x axis: CDR3 length (base pair size). (B) Hierarchical cluster analysis of CDR3 lengths from 20 *TRβV* groups and 1 subgroup from the proximal jejunum, distal jejunum and spleen from pigs at 21 days of age.

Having demonstrated that our novel microbiota expanded the CD4 T-cell compartment, we investigated whether this expansion altered TCR Vβ repertoire by examining complementarity determining region 3 (CDR3 length) within 20 *TRβV* groups and 1 *TRβV* subgroup (Table S1). Preliminary visual inspection of electropherograms indicated that spectratypes generated from splenic tissues typically demonstrated a Gaussian distribution pattern, whereas spectratypes generated from the proximal and distal jejunum clearly showed skewed distribution patterns (Figure 3A). These findings are in concordance with our previous studies in rats [30], where examination of CDR3 length plots clearly demonstrated a more restricted repertoire in both the proximal and distal jejunum in comparison to the spleen. Following visual inspection, the peak height data contained within individual spectratypes generated for each *TRβV* group/subgroup and each tissue sample was extracted and compiled. These data were then standardised, so that within each spectratype the proportion that each peak height contributed to the total spectratype was calculated. As each peak is a proportion of the total, when this data is plotted the height of peaks also relate to peak spread. Thus, a peak height of 1 represents a highly restricted repertoire with a single CDR3 length. The standardised data was analysed by hierarchical cluster analysis (Figure 3B). This confirmed the pattern that was clear on visual inspection: based on TCR Vβ repertoire, spleens from different pigs (regardless of treatment) clustered together, and a more restricted T-cell repertoire was recruited to both the proximal and distal jejunum in comparison to the solid lymphoid tissue from the spleen. However, the cluster analysis also demonstrated a random distribution of GF and colonised pigs through the hierarchy, and provided no evidence that the diversity of the TCR Vβ repertoire in the intestine was affected by colonisation (Figure 3B). If colonisation had influenced repertoire, the expectation would be that the GF pigs would cluster together based on colonisation status: this was clearly not the case. This was confirmed by PCA of CDR3 lengths followed by GLM for all *TRβV* groups/subgroup. PCA allows a reduction in the number of dimensions of a dataset, transforming a set of possible correlated variable into a group of uncorrelated variables (principle components). This is a useful technique for the analysis of spectratype data where large numbers of different possible CDR3 lengths may exist within each *TRβV* group/subgroup. Having generated the uncorrelated principle components, it is then possible to use GLM to examine the effects of factors such as colonisation status, and tissue on each component. Whilst there was a significant effect of tissue (spleen or jejunum) on principal components 1 and 4 (p<0.01), there was no effect of status (GF or colonised) on any of the principle components (Table 2), indicating that colonisation status did not affect TCR Vβ repertoire in the intestine.

**Table 2:** T-cell repertoire is different between tissues but not between GF and colonised pigs.

Principal components					
	1	2	3	4	5
**Corrected model**	0.006	N/S	N/S	0.001	N/S
**Status**	N/S	N/S	N/S	N/S	N/S
**Tissue**	0.004	N/S	N/S	0.0003	N/S
Status*tissue	N/S	N/S	N/S	N/S	N/S

Principle component analysis of CDR3 lengths for all V beta families was performed for a maximum of 25 iterations for convergence using an un-rotated factor solution. Table 2 shows the p-values from a fully factorial general linear model of the first five principal components with colonisation status (GF or colonised) and tissue as fixed factors. A p-value of <0.01 was considered significant using a Bonferroni correction for 5 tests.

### Colonisation alters mucosal APC subsets

Given the previous studies indicating that microflora may affect APC subsets in different ways in mice and pigs [17], [29], and the observation that DCs are able to make direct contact with the intestinal microbiota in the mouse intestine [12], [13], we hypothesised that colonisation would result in early recruitment of specific subsets of APC rather than act on all subsets.

**Figure 4:**
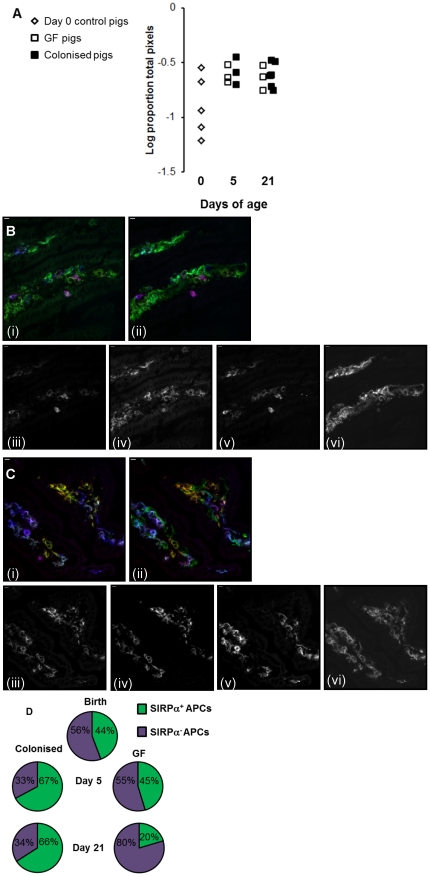
Colonisation affects mucosal MHCII^+^ APC subsets. (A) Area of MHCII^+^ cell staining in jejunal sections from piglets at birth (open diamonds) and GF piglets (open squares) and colonised piglets (black squares) at 5 and 21 days of age (see Table 1 for numbers of piglets in each experiment). (B) SIRPα staining in jejunal mucosa from a GF pig and (C) a colonised pig at 21 days of age. (B) (i) and (C) (i) show staining with antibodies to CD16 (red), SIRPα (green) and CD11R1 (blue). (B) (ii) and (C) (ii) show the same sections with the same cells in red and blue but with MHCII DR in green. In both (B) and (C), (iii) shows the red channel (SIRPα); (iv) shows the green channel (CD16), (v) shows the blue channel (CD11R1); (vi) shows the infra-red channel (MHC class II). Scale bar represents 10 µm. (D) Partitioning of MHCII between SIRPα^+^ (green shading) and SIRPα^−^ (purple shading) APC subsets in the intestines of pigs at birth and from both treatment groups at 5 days and 21 days of age (see Table 1 for numbers of piglets in each experiment).

**Figure 5:**
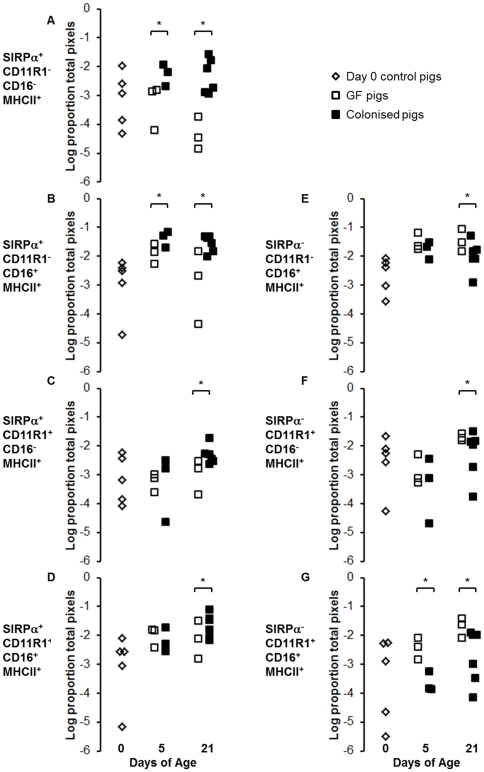
Colonisation selectively increases SIRPα^+^ APC subsets within the first five days of life. Intestinal APC (MHCII^+^) subsets were defined by their expression of SIRPα, CD11R1 and CD16. (A–D) show SIRPα^+^ subsets (E–G) show SIRPα^−^ subsets from piglets at birth (n = 5; light grey bars) and GF piglets (white bars) and colonised piglets (dark grey bars) at 5 (n = 3 GF and n = 3 colonised) and 21 (n = 4 GF and n = 6 colonised) days of age (see Table 1 for numbers of piglets in each experiment). Error bars represent standard errors of the mean. After a Bonferroni correction was applied for multiple tests (ANOVA), a p-value of <0.003 was considered to be significant.*p<0.003. The MHCII^+^ SIRPα^−^ CD11R1^−^CD16^−^ subset is not shown as this represents endothelial MHCII, which was unaffected by colonisation or age.

Since the majority of myeloid APCs reside in the villi of the pig small intestine, we used immunofluorescence to examine MHCII expression on the two main groups of APC in pig intestinal villi (myeloid (professional, potentially migratory) APCs and endothelial APCs). Several subsets of myeloid APC have been described in the pig [14], [20], and MHCII is constitutively expressed, as in humans, on porcine intestinal capillary endothelium. Examination of the villi but not the crypts meant that B-cell associated MHCII was excluded from the analysis. The expression of MHCII in the intestinal mucosa increased with age in both colonised and GF pigs. Strikingly, there was no effect of colonisation on total MHCII expression (Figure 4A). However, analysis of immunohistology images indicated that, whilst colonisation did not affect total MHCII expression, it did alter partitioning of the MHCII between different APC subsets (Figure 4B and C). Quantitative image analysis showed no difference in MHCII expression on endothelium (data not shown). However, examination of the migratory APC subsets demonstrated a clear effect on partitioning of MHCII between SIRPα^+^ and SIRPα^−^ APCs (Figure 4D). At birth, MHCII was split roughly equally between SIRPα^+^ and SIRPα^−^ APC subsets. At 5 days, this situation persisted in GF pigs, whereas colonisation increased the amount of MHCII associated with SIRPα^+^ APCs. Further analysis showed that this was due to an increase in the SIRPα^+^CD11R1^−^ population but not the SIRPα^+^CD11R1^+^ population (p<0.003; Figure 5 A–D). However, by 21 days, colonisation increased all subsets of SIRPα^+^ APCs (SIRPα^+^CD11R1^−^ and SIRPα^+^CD11R1^+^; p<0.003; Figure 5 A–D). Conversely, in the GF controls at this time point, MHCII was now preferentially associated with SIRPα^−^ APC subsets (Figure 4D) due to an increase in SIRPα^−^CD11R1^+^ APCs and a decrease in SIRPα^+^CD11R1^−^ APCs (p<0.003; Figure 5 E–G).

## Discussion

The importance of the interaction between the intestinal microbiota and the immune system is becoming increasingly clear [50]. It is widely postulated that the intestinal microbiota may not only impact on active immune responses within the gut, but that changes in the microbiome may also contribute to the increasing incidence of allergic disease [9], [51]. Therefore, development of models in which this interaction can be studied in neonates are central to understanding the mechanisms by which the microbiota impacts on immune development and, in the long term, to developing therapies based on manipulation of the microbiota. In this study, we describe a highly inbred GF pig model that prevents contact of the piglets with the sow, controlling for the impact of maternal factors on immune development. It should be noted that the piglets received no vitamin supplementation, potentially impacting on the development of the immune system. For example, the vitamin K-dependent protein Gas6 has been shown to be important for immune regulation: incubation of dendritic cells with Gas6 decreases their proinflammatory cytokine production [52]. In addition, the influence of vitamin A on intestinal homeostasis is well documented [53], [54]. Nevertheless, we are able to show that the microbiota used in these experiments expands the immune system in neonates over a time course comparable to that observed in conventionally reared piglets [55] demonstrating that, whilst this is a reductionist model, it is also applicable to the colonisation process in conventional, neonatal animals.

Importantly, our results clearly demonstrate early, differential effects of colonisation on different APC subsets in neonatal animals. The data suggest that the expression of SIRPα on mucosal APCs was critically regulated by colonisation, whereas the expression of CD11R1 increased with age, independent of colonisation. Previous studies in pigs and rodents have correlated the expression of SIRPα by intestinal APCs with specific functions. In pigs, the expression of this molecule has been associated with migration from the mucosa to the lymph nodes [20], although we have not examined expression of SIRPα in the mesenteric lymph nodes in the studies described here. In mice, studies have demonstrated that the SIRPα^+^CD103^−^ APC subset may be involved in the generation of inflammatory bowel disease, inducing a Th17 response both *in vitro* and *in vivo* [23]. In the work presented here, there was a clear increase in SIRPα^+^ APCs alone as early as 5 days after colonisation. Notably, MHCII expression in the intestinal villi (and hence APC number) appeared unaffected by colonisation, indicating that the increase in SIRPα^+^ APCs was at the expense of SIRPα^−^ APCs. Since immunohistology measures areas of cells as a proportion of total area, rather than of total cells (as in flow cytometry), the most likely explanation for the change is an upregulation of the molecule on previously SIRPα^−^ APCs. However, whilst our preferred hypothesis is that APC subsets within the mucosa are highly plastic at this age, it is also possible that SIRPα^+^ and SIRPα^−^ APCs are generated from separate precursors and that the increase in SIRPα^+^ APCs reflects a combination of two independent effects: an increased retention/recruitment of these cells combined with decreased retention/recruitment of SIRPα^−^ APCs. This would be supported by studies in mice demonstrating generation of intestinal DC subsets from different precursors [16].

Rodent studies have indicated that the SIRPα^−^CD47 interaction is required for migration to the lymph nodes [23]. Therefore, the upregulation of SIRPα demonstrated here in response to colonisation may reflect an increased need for antigen sampling and subsequent migration by APCs in the colonised pigs. The increased ability of APCs from the colonised pigs to migrate to organised lymphoid tissue and prime naïve CD4^+^ T-cells would result in a later increase in CD4^+^ T-cells in the mucosa as observed in this study: while the impact of colonisation on APC subsets was clear by 5 days after birth, the effect on CD4^+^ T-cells was not apparent until 21 days. However, previous studies by our group and others have demonstrated that antigen presentation may also occur directly in the intestinal mucosa in pigs and humans [14], [56]. Therefore, a role for SIRPα^+^ APCs in directing immune responses *in situ* in the mucosa cannot be ruled out.

Expansion of the lymphoid component of the intestinal mucosa has previously been demonstrated in mice and pigs [33], [34], [39] and we have confirmed this observation in the study described here. Interestingly, our results also indicate that both plasma cells and CD4 T-cells in the intestine increased between pigs at day 0 and day 5. The day 0 pigs were caesarean derived, 48 hrs before a natural birth was due. As such, they represent fetal pigs in terms of their development rather than neonatal animals. We have previously observed a population of lymphocytes in the intestines of foetal piglets that is not present in neonatal animals (unpublished data). This observation is reinforced by the results presented here. We hypothesise that the fetal cortisol ‘spike’ associated with the process of parturition may result in the death of these cells or of their movement from the mucosa to the periphery. Clearly, this observation requires further investigation.

We have extended the previous studies in pigs to show that TCR Vβ repertoire diversity is unaffected by expansion of the T-cell compartment, although we cannot exclude the possibility that this expansion may have masked more subtle repertoire skewing. Due to the limitations of the spectratyping technique, relatively rare clones (that may, nevertheless, be important for microbiome-host homeostasis) may be below the level of detection or obscured by other more dominant clones within a *TRβV* group. In addition, it should be noted that if two T-cells share the same CDR3 length, this does not necessarily mean that they have the same TCR sequence: i.e. the same length of CDR3 does not necessarily equate to the same sequence and therefore spectratyping does not discriminate between different clones that have the same CDR3 length. Moreover, analysis of TCR Vβ chain alone represents only a portion of the total TCR repertoire diversity present within the T-cell population.

Similar, non-specific expansion of B-cell repertoire has been proposed from studies in SFB monocolonised mice in which only a small proportion of the IgA produced was SFB-specific [33], [34], and with studies in pigs indicating that production of immunoglobulin in response to colonisation is antigen non-specific [31], [32]. However, more recent experiments carried out in a reversible GF colonisation system in mice have suggested that IgA production was specific for the colonising microbiota (specifically *E. coli* HA107) [57]. It is possible that this is a consequence of the effects of different colonising bacteria on stroma as well as on immune cells. For example, the preferential induction of classical mitogenic cytokines (such as IL-7 and IL-15) by SFB but not by *E. coli* HA107 may lead to an antigen non-specific expansion in the former system but not in the latter. However, a recent paper has also indicated that commensal antigen-specific peripherally-induced regulatory T cells are generated in response to colonisation [58]. Clearly, further work is required to elucidate the reasons for these apparently opposing sets of data, since polyclonal expansion of T- and B-cell repertoire by microbial colonisation would be likely to affect responses to unrelated antigens (pathogens or food) to a much greater extent than caused by antigen specific responses to microbiota. Whilst the data presented here demonstrates that colonisation does not influence repertoire in the gut, the intestinal repertoire was nevertheless skewed in comparison to the repertoire in the spleen. Previous work by our group has indicated that this is also the case in the rat [30] and the data is also supported by mouse studies demonstrating an oligoclonal repertoire in the gut [59]. Thus, the intestinal environment clearly promotes either the recruitment or retention of specific T cell clones. Since there was no apparent difference between germ-free and colonised piglets in recruited repertoire, this selection may be largely driven by food-derived, non-microbial antigens: a comparison of the effects of diets containing different antigens in a GF system would go some way to addressing this question.

In this study we have used a piglet model to study the effects of defined microbial colonisation on development of the immune system in early neonates. Our results suggest a defined sequence of events in immune development after colonisation. Firstly, microbial colonisation drives the recruitment of SIRPα^+^ APCs to the intestine, while SIRPα^−^CD11R1^+^ APCs expand with age regardless of colonisation status; secondly, colonisation recruits CD4^+^ T cells to the mucosa without altering the repertoire of the T-cell population. We propose that this T-cell recruitment is directly driven by the early recruitment of SIRPα^+^ APC subsets, and that the T-cells then co-operate with APCs and stroma to promote class-switching and plasma cell differentiation. Thus, we propose that the critical interaction in neonates is between SIRPα^+^ APCs and factors in the microbiota, and that this occurs very quickly after colonisation begins. Thus, any manipulation of this interaction would have to occur in the immediate post natal period in conventional animals where the colonisation process starts at birth.

## Supporting Information

Table S1 TRβV group/subgroup specific primer sequences used for spectratyping. All PCR reactions had the same reverse primer, Universal C*β*. Twenty *TRβV* group, and 1 *TRβV* subgroup-specific forwards primers are listed. ‘S’ added after the V*β* group indicates designation according to the ImMunoGeneTic (IMGT) system [39].
(DOC)
