# Supplementary material for: Neonatal Colonisation Expands a Specific Intestinal Antigen-Presenting Cell Subset Prior to CD4 T-Cell Expansion, without Altering T-Cell Repertoire
Source: PLoS One. 2012 Mar 19;7(3):e33707. doi: 10.1371/journal.pone.0033707 (PMC3307746; doi:10.1371/journal.pone.0033707)
Supplement: Table S1 — TRβV group/subgroup specific primer sequences used for spectratyping. All PCR reactions had the same reverse primer, Universal Cβ. Twenty TRβV group, and 1 TRβV subgroup-specific forwards primers are listed. ‘S’ added after the Vβ group indicates designation according to the ImMunoGeneTic (IMGT) system [39]. (DOC) [file pone.0033707.s001.doc]

# *Table S*1.

|  | **Primer sequence (5’- 3’)** |
| --- | --- |
| **Universal *Cβ*** | ATCTCCGCTTCCGATGGT |
| ***TRβV* group/subgroup** |  |
| ***TRβV*2S** | GGGAGTAGGCCACATGGAAC |
| ***TRβV* 3S** | GGTCCCTAAAATGTGAGCAAAAG |
| ***TRβV* 4S** | CAGATACCTGGTCCTGGGAA |
| ***TRβV* 5S** | CACCGAGACATCTGATTAAAGC |
| ***TRβV* 6S** | ACTGAACCGTGCCCAAGA |
| ***TRβV* 7S** | TCTGAGCTGAAATTGCTCTCC |
| ***TRβV* 9S** | AGCTTTTGTCTCCACAGGTCA |
| ***TRβV* 10S** | CCTGTGATGTTGGCATCCTT |
| ***TRβV* 11S** | TGTTTCTCAGTTGCCCCAGA |
| ***TRβV* 12S** | CACCCAGACACGAGGTGA |
| ***TRβV* 12-AS** | CAACAACGGGTCTCCTGTG |
| ***TRβV* 15S** | ACCCTTCTTTCTGTTCCCTTG |
| ***TRβV* 19S** | CCCAGGACAAGGACTGAGA |
| ***TRβV* 20** | CCCCTGCTGATAGCAACTTC |
| ***TRβV* 21** | CGAGTGCCTCAAGAACTCCT |
| ***TRβV* 24** | CCCAGATCCCAAGGAACAAG |
| ***TRβV* 25** | CGGGGTTAATTCCACAGAG |
| ***TRβV* 27** | TCAAGGAACGTTGATTTGGT |
| ***TRβV* 29** | ACCGTCAGCTTCTAGGACAAAG |
| ***TRβV* 30** | TGACCAGAAAGATCCTGAAAAG |
| ***TRβV* XS** | AGGCCACATCCCTTCCT |
